# Lysosomes in T Cell Immunity and Aging

**DOI:** 10.3389/fragi.2021.809539

**Published:** 2021-12-09

**Authors:** Jun Jin, Huimin Zhang, Cornelia M. Weyand, Jorg J. Goronzy

**Affiliations:** ^1^ Department of Immunology, Mayo Clinic, Rochester, MN, United States; ^2^ Department of Medicine/Rheumatology, Mayo Clinic, Rochester, MN, United States; ^3^ Department of Medicine, Stanford University, Stanford, CA, United States

**Keywords:** lysosome, T cell aging, late endosomes, mTORC1, memory T cell, T follicular helper cell

## Abstract

Lysosomes were initially recognized as degradation centers that regulate digestion and recycling of cellular waste. More recent studies document that the lysosome is an important signaling hub that regulates cell metabolism. Our knowledge of the role of lysosomes in immunity is mostly derived from innate immune cells, especially lysosomal degradation-specialized cells such as macrophages and dendritic cells. Their function in adaptive immunity is less understood. However, with the recent emphasis on metabolic regulation of T cell differentiation, lysosomes are entering center stage in T cell immunology. In this review, we will focus on the role of lysosomes in adaptive immunity and discuss recent findings on lysosomal regulation of T cell immune responses and lysosomal dysfunction in T cell aging.

## Introduction

Ever since they were discovered by Christian de Duve in 1955 (De Duve et al., 1955), lysosomes have been known as acidic terminal degradation compartments that are essential to maintain proteostasis (De Duve, 2005), thereby also functioning in cellular quality control (Lawrence and Zoncu, 2019). They exert quality control through numerous cellular processes, including degradation and nutrient recycling, clearance of damaged cellular components, elimination of intracellular and extracellular pathogens, and exocytosis (Perera and Zoncu, 2016; Lawrence and Zoncu, 2019; Ballabio and Bonifacino, 2020). Until the early 2000s, their role in waste or pathogen scavenging and nutrient recycling has dominated our thinking. A major new orientation in this field was the discovery that lysosomes are the platform for the activation of mTORC1 (mechanistic target of rapamycin complex 1), a master regulator coordinating cell growth and cellular metabolism with environmental inputs, thereby facilitating the switch to anabolic metabolism, which is required for cell proliferation and differentiation (Sancak et al., 2010). The lysosome therefore functions as a signaling hub, sensing the cellular nutrient and energy state and controlling the switch between anabolism and catabolism (Kim and Guan, 2019).

The discoveries of the lysosome as a degradation center and subsequently as an mTORC1 signaling platform have expanded our current knowledge on lysosomal regulation of innate immunity. Dendritic cells and macrophages largely rely on the powerful lysosomal digestion system to eliminate invaded pathogens and process the antigen for antigen presentation (Ballabio and Bonifacino, 2020). The roles of lysosomes in T cells, which do not specifically rely on lysosomal degradation in their effector functions, are only beginning to be uncovered. Here, we give an overview of lysosomal functions and then focus on their roles in T cells, with particular emphasis on lysosomal defects in older adults.

## Overview of Lysosomal Function

### Degradation and Recycling

**FIGURE 1:**
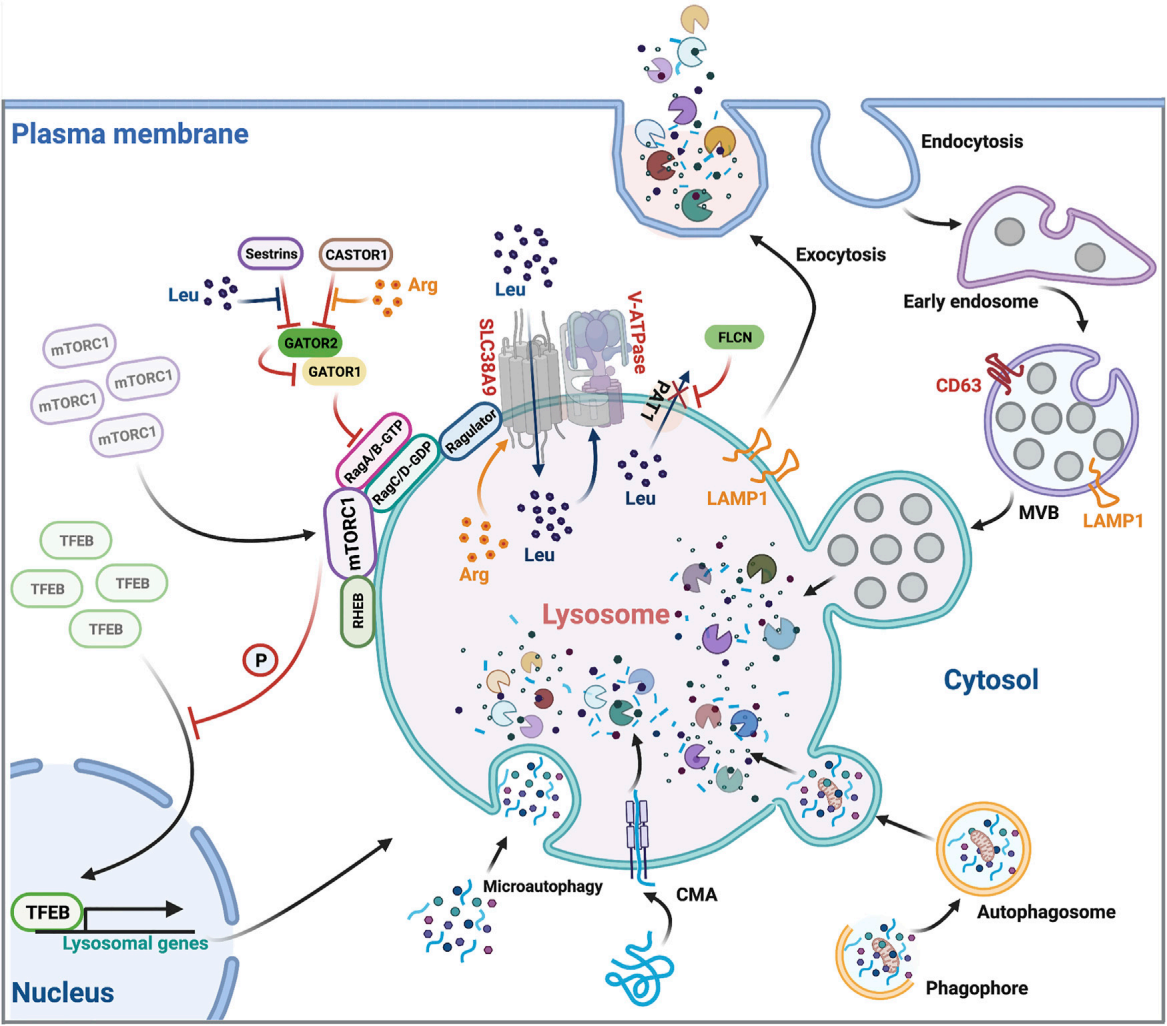
Overview of lysosomal function. Lysosomes are involved in many cellular processes including endocytic protein degradation, autophagic protein degradation, exocytosis, and regulating mTORC1 signaling. They cross-regulate themselves in a feedback loop involving mTORC1-dependent TFEB phosphorylation, TFEB nuclear translocation and transcription of lysosomal genes. Leu, Leucine; Arg, Arginine.

Lysosomes have a powerful digestion system, containing approximately 60 hydrolases in its lumen, including proteases, phosphatases, lipases, sulfatases and nucleases (Bonam et al., 2019). These hydrolytic enzymes are capable of degrading misfolded and damaged proteins, protein aggregates, damaged organelles as well as lipids, nucleic acids, carbohydrates and even intracellular pathogens (Settembre et al., 2013) (Figure 1). To avoid these hydrolases leaking into the cytosol and causing unintended damage, they are sealed by the limiting lysosomal membrane that is enriched for lysosome-associated membrane protein 1 (LAMP1) (Saftig and Klumperman, 2009). LAMP1 is heavily glycosylated on its luminal side and forms the glycocalyx, a barrier protecting the limiting membrane from autodigestion by the hydrolytic enzymes within the lysosomal lumen (Saftig and Klumperman, 2009; Perera and Zoncu, 2016). Of note, these enzymes only exhibit a highly active hydrolytic activity in a low pH environment (pH 4.5–5.0) (Mindell, 2012), a typical property of the lysosomal lumen. The proton pump, vacuolar adenosine triphosphatase (V-ATPase), is essential for transporting cytosolic protons into the lysosomal lumen and therefore maintaining an acidic lumen environment for optimal hydrolase activities (Mindell, 2012). Lysosomal cargos are mostly sequestered from two sources: extracellular and surface materials via endocytosis and intracellular materials via autophagy (Compeer et al., 2018; Macian, 2019) (Figure 1). During endocytosis, the plasma membrane invaginates to form endocytic vesicles enclosing extracellular or cell surface cargos, which then fuse with early endosomes. Subsequently, early endosomes mature into multivesicular bodies (MVBs), also termed late endosomes, which then fuse with the terminal lysosomes and form a hybrid endolysosome, where degradation ensues (Luzio et al., 2007) (Figure 1). During macroautophagy, cytoplasmic materials, including damaged organelles such as mitochondria and insoluble protein aggregates, are targeted for entrapment by the phagophore, which then forms the autophagosome, a double-bilayer membrane structure (Li and Zhang, 2020). Autophagosomes mature and ultimately fuse with lysosomes to become autolysosomes, where the degradation occurs (He et al., 2012) (Figure 1). Soluble cytosolic proteins can also be degraded through microautophagy and chaperone-mediated autophagy (CMA), other forms of autophagy, in which the cargos directly fuse with the lysosome without formation of phagophore and autophagosome (Macian, 2019) (Figure 1). After cargos are degraded in lysosomes, nutrients are recycled back to the cytosol through diverse transporters located in the lysosomal membrane including solute carriers that export amino acids, sugars and nucleosides, and transporters that export cholesterol and lipids (Ballabio and Bonifacino, 2020).

### Exocytosis

Beyond cargo degradation and nutrient recycling, lysosomes are also capable of fusing with the plasma membrane, leading to the extracellular release of lysosomal content (Medina et al., 2011) (Figure 1). This lysosomal exocytosis contributes to the plasma membrane expression of the endolysosomal protein LAMP1, predominantly used to assess the level of lysosomal exocytosis (Andrews, 2017). The transcription factor EB (TFEB), an important regulator of lysosomal exocytosis, increases the pool of lysosomes in the proximity of the plasma membrane, and promotes their fusion with the membrane through activating the lysosomal Ca^2+^ channel Mucolipin TRP Cation Channel 1 (MCOLN1) and raising intracellular Ca^2+^ levels (Medina et al., 2015). The role of lysosomal exocytosis is not just for waste disposal but also involves many other cellular processes including antigen presentation (Yuseff et al., 2011), lytic granule release (Miyatake et al., 2018) and plasma membrane repair (Reddy et al., 2001).

### Signaling Platform

Leucine and arginine are the most important two nutrients that stimulate and maintain mTORC1 activities on the lysosomal surface (Wolfson and Sabatini, 2017; Takahara et al., 2020). Cytosolic, free leucine and arginine coordinate with lysosomal luminal leucine and arginine to regulate mTORC1 activation and signaling by an inside-out mechanism (Zoncu et al., 2011) (Figure 1). Briefly, when lysosomal luminal arginine is enriched, it promotes lysosomal uptake of cytosolic leucine through the lysosomal leucine transporter SLC38A9 (Wang et al., 2015). Concurrently, Proton-coupled amino acid transporter 1 (PAT1), another lysosomal amino acid transporter, is excluded from lysosomes by Folliculin (FLCN), which prevents leucine from exiting the lysosome, leading to a massive accumulation of leucine within the lysosomal lumen (Wu et al., 2016). High-level luminal leucine promotes the activity of lysosomal membrane proton transporter V-ATPase. Activated V-ATPase then interacts with the scaffolding complex Ragulator, recruiting the Rag heterodimers (RagA/B heterodimerized with RagC/D) to the lysosomes, thereby mobilizing cytosolic inactivated mTORC1 to the lysosomal membrane (Zoncu et al., 2011; Milkereit et al., 2015) (Figure 1). Lysosomal resident mTORC1 now meets its activator, another lysosomal membrane protein Ras homolog enriched in brain (RHEB) that not only facilitates the mTORC1 substrate recruitment but also promotes the kinase activity of mTORC1 (Yang et al., 2017) (Figure 1). To sustain high mTORC1 activity, cytosolic leucine and arginine bind to and inhibit sestrins (Wolfson et al., 2016) and cytosolic arginine sensor for mTORC1 subunit 1 (CASTOR1) (Chantranupong et al., 2016). The release of their inhibitory effects allows the multisubunit protein complex GTPase-activating protein activity toward Rags 2 (GATOR2) to bind and antagonize GATOR1. GATOR1 inactivation promotes GTP loading on RagA/B, facilitates mTORC1/Rag heterodimer formation, and sustains mTORC1 signaling (Bar-Peled et al., 2013) (Figure 1). Taken together, lysosomes are essential for mTORC1 signaling through providing the amino acid resources and serving as the platform equipped with the necessary proteins for mTORC1 activation. Conversely, mTORC1 inhibits lysosome biogenesis and activity through a TFEB-dependent negative feedback loop (Roczniak-Ferguson et al., 2012). TFEB is a master transcription factor for lysosomal biogenesis (Settembre et al., 2011). When lysosomal activity is deficient, mTORC1-dependent phosphorylation of TFEB is reduced, resulting in the translocation of TFEB into the nucleus, where it stimulates the transcription of lysosomal genes and restores lysosomal activities. Likewise, adequate lysosomal activities trigger the mTORC1-dependent phosphorylation and cytoplasmic retention of TFEB and the termination of its transcriptional induction (Settembre et al., 2011) (Figure 1). This lysosome-to-nucleus signaling allows the lysosome to adapt to the cellular metabolic state through regulating transcription of its constituents.

## Roles of Lysosomes in T Cells

### Exertion of Cytotoxicity

**FIGURE 2:**
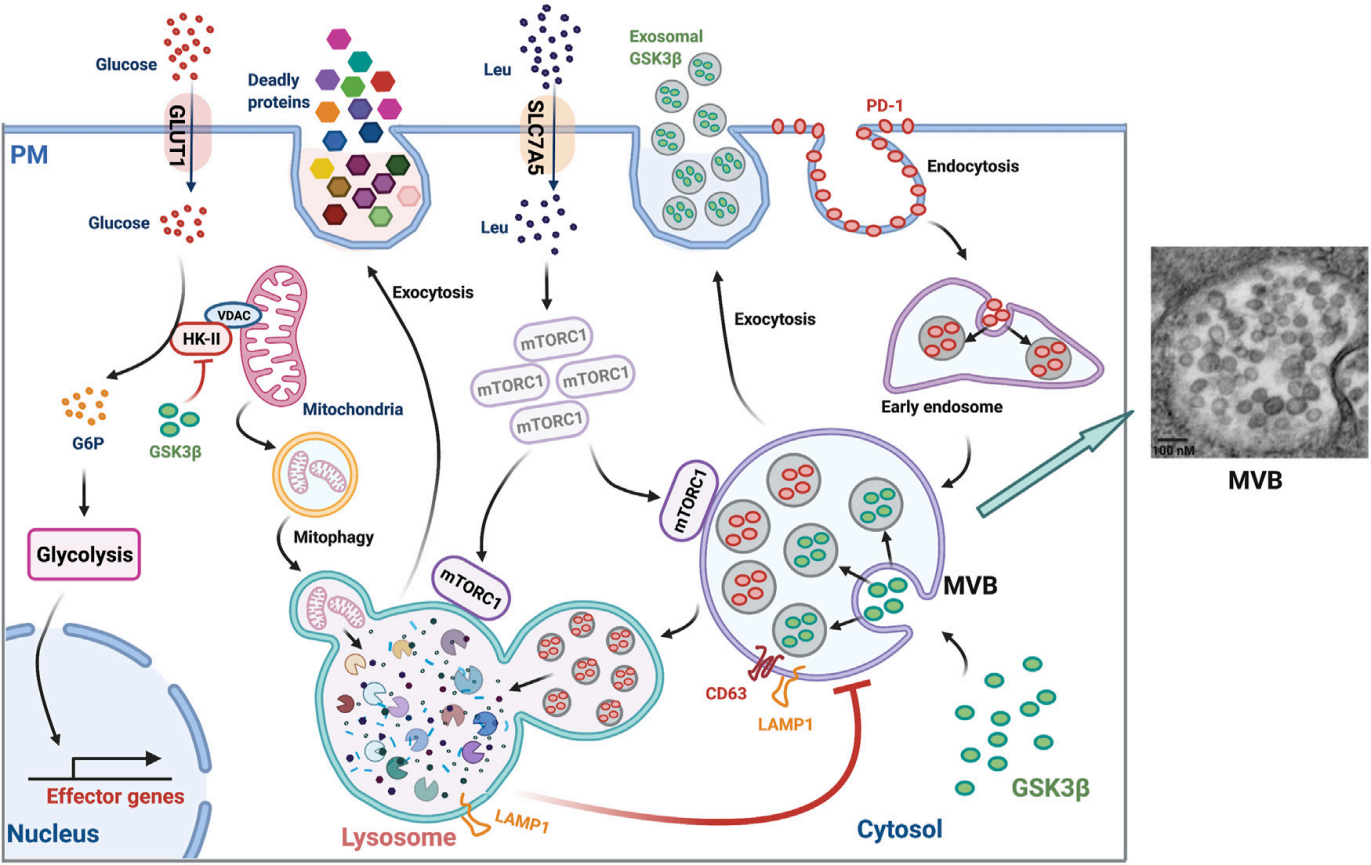
Lysosomal function in T cells. **(Left panel)** Lysosomes play many roles in T cells: 1) degrading PD-1, 2) regulating MVB turnover and MVB sequestration and exosomal release of GSK3β, 3) supporting late endosomal activation of mTORC1, 4) regulating cell surface leucine transporter (SLC7A5) expression, 5) releasing lethal proteins, 6) maintaining mitochondria fitness by promoting mitophagy, 7) regulating glycolysis and subsequent effector gene expression by modulating cytosolic GSK3β-dependent mitochondria HK-II levels. PM, plasma membrane; G6P, Glucose 6-phosphate. **(Right panel)** Transmission electron microscopy of an MVB in an *in vitro* day 3-stimulated naïve T cells showing the existence of dozens of vesicles with a size ranging from ∼50 to ∼100 nm inside the lumen (imaged by Jin et al.). MVBs are enriched in T cell responses of older adults (Jin et al., 2020).

Lysosomal exocytosis of lytic granules or degranulation is mediated by a subset of lysosomes, termed “secretory lysosomes” (Blott and Griffiths, 2002; Bossi and Griffiths, 2005). They are quite similar to conventional lysosomes but differ in their luminal contents (Saftig and Klumperman, 2009). In cytotoxic CD8^+^ T cells, secretory lysosomes contain lethal proteins including perforin and granzymes (Peters et al., 1991; Martinez-Lostao et al., 2015) in addition to conventional lysosomal hydrolases (Figure 2). Upon T cells interacting with target cells, apoptosis is induced by perforin, which generates pores in plasma membranes, allowing granzymes to enter the target cells (Lettau et al., 2015), such as virus-infected cells (Rosendahl Huber et al., 2014) and cancer cells (Durgeau et al., 2018). Plasma membrane LAMP1 is frequently used as a functional marker for cytotoxicity of CD8^+^ T cells (Alter et al., 2004). Interestingly, LAMP1 also protects cytotoxic cells from self-destruction during lysosomal degranulation, recapitulating its function in preventing lysosomal hydrolases from leaking into the cytosol (Cohnen et al., 2013). How lysosomes acquire secretory activity remains poorly understood. Taken together, lysosomal exocytosis of lytic granules plays an important role in immuno-surveillance and host defense against cancer and viral infections.

### Degradation of the Program Cell Death Protein 1 (PD-1)

The immune checkpoint inhibitory receptor PD-1 is highly expressed in activated T cells and is involved in the inhibition of immune responses (Patsoukis et al., 2020). As a plasma membrane receptor, PD-1 is internalized upon ligation with its ligands PD-L1 and PD-L2 and sorted into early endosomes (Wang et al., 2019). Endosomal PD-1 is ultimately sequestered into the terminal lysosome for degradation (Jin et al., 2021) (Figure 2), documenting an important role of lysosomal degradation in regulating PD-1 protein turnover. Correspondingly, improved lysosomal degradation of PD-1 reduced its cell surface expression and enhanced proliferative responses of primary human T cells to pertussis and SARS-CoV-2 peptide stimulation *in vitro* (Jin et al., 2021). *In vivo*, enhanced lysosomal degradation of PD-1 in LCMV-specific T cells improved T cell expansion by at least three-fold at peak response after LCMV infection. Consequently, germinal center responses, CD8^+^ memory T cell generation, and recall responses to infection were improved (Jin et al., 2021). Thus, boosting lysosomal activity to reduce PD-1 expression is a promising strategy to enhance T cell immunity. However, in some settings, endocytosed PD-1 undergoes ubiquitination and proteasomal degradation, making lysosomal degradation oblivious (Meng et al., 2018; Serman and Gack, 2019). FBXO38, an E3 ubiquitin ligase, mediates ubiquitination and subsequent proteasomal degradation of endocytosed PD-1 (Meng et al., 2018). Mice bearing conditional knockout of Fbxo38 in their T cells had faster tumor progression due to high levels of PD-1 expression in tumor-infiltrating T cells (Meng et al., 2018). In addition, internalized endosomal PD-1 was reported to be sorted into recycling endosomes that recycled PD-1 back to the cell surface and therefore maintained its surface abundance (Wang et al., 2019). This was typically observed in exhausted tumor-infiltrating T cells, where high levels of TOX facilitated the endocytic recycling of PD-1 (Wang et al., 2019). Overall, lysosomal sequestration and degradation constitutes an important regulatory arm of cell surface PD-1 expression and the corresponding immune checkpoint in T cell responses.

### MVB Turnover and Exosome Secretion

The MVB or late endosome is an acidic organelle with no luminal hydrolytic enzymes and thus no proteolytic activities but containing multiple intraluminal vesicles (ILVs) that harbor cargos (Piper and Katzmann, 2007; Huotari and Helenius, 2011) (Figure 2). In general, the MVB has two fates: fusion with the terminal lysosome to form an autolysosome for cargo degradation and recycling; and fusion with the plasma membrane and release of its luminal vesicles as exosomes into the extracellular environment (Piper and Katzmann, 2007; Cullen and Steinberg, 2018). The choice between lysosome vs. plasma membrane fusion is poorly understood. We recently found that the terminal lysosomal activity is an important regulator of MVB turnover and MVB release of exosomes in T cells (Jin et al., 2020). Lysosome inhibition by a pharmacological compound (leupeptin or bafilomycin A1) or genetic silencing of TFEB in activated T cells induced intracellular accumulation of LAMP1-positive compartments representing late endosomes (Jin et al., 2020) (Figure 2). Concurrently, the lysosome-inhibited T cells exhibit an increased number of acidic organelles with reduced proteolytic activity (Jin et al., 2020). Enlarged intracellular structures coexpressing surface LAMP1- and CD63 corroborated that MVBs were expanded. Concomitantly, the trafficking route of MVBs was shifted from lysosomal to plasma membrane fusion in lysosome-inhibited T cells, resulting in increased secretion of exosomes and increased cell surface expression of CD63 (Verweij et al., 2018), a surrogate marker of MVB fusion with the plasma membrane. The exosomes secreted from lysosome-inhibited cells abundantly contained the cytotoxic protein granzyme B that harms cells in the local environment including B cells (Jin et al., 2020). Taken together, optimal lysosomal activity and lysosomal fusion with MVBs is essential for the control of MVB turnover and the maintenance of a healthy microenvironment of T cells.

### Regulation of T Cell Size and Differentiation by GSK3β Sequestration

As discussed above, lysosomes regulate the turnover and trafficking of MVBs. Lysosome inhibition induces an expansion of MVBs. Expansion of the MVB compartment induces the sequestration of glycogen synthase kinase 3β (GSK3β) into the intralumenal vesicles of MVBs and thus enhances exosomal release of GSK3β into the extracellular fluid, resulting in a loss of the intracellular GSK3β protein in lysosome-inhibited T cells (Jin et al., 2020). Consequently, GSK3β-associated activities are compromised, such that intracellular protein turnover is suppressed, leading to an increase in cell size. Glycolytic activity is enhanced, which promotes effector cell differentiation with increased production of the effector protein granzyme B, perforin and B-lymphocyte-induced maturation protein 1 (BLIMP1) (Jin et al., 2020) (Figure 2). Mechanistically, GSK3β reduces the half-life of about 20% of total cellular proteins by promoting phosphorylation-dependent protein ubiquitination and subsequent proteasomal degradation (Taelman et al., 2010). Furthermore, MVB sequestration of GSK3β induces an accumulation of hexokinase II (HK-II) at the mitochondria, thereby enhancing glycolysis manifested as an increased glucose uptake and lactate secretion (Jin et al., 2020) (Figure 2). Enhanced glycolysis favors T cell differentiation into granzyme B–expressing effector cells instead of memory cells (Jin et al., 2020). Consistent with our findings in human CD4^+^ T cells, granzyme B production and cytotoxicity in CD8^+^ T and NK cells are increased following GSK3β inhibition under both *in vitro* and *in vivo* conditions (Taylor et al., 2016; Cichocki et al., 2017). Impaired lysosomal function diverts T cell differentiation toward pro-inflammatory subsets and exacerbates the *in vivo* inflammatory response (Baixauli et al., 2015). However, the role of GSK3β in regulating effector vs. memory differentiation is far more complex. A previous study showed that pharmacological GSK3β inhibition increased β-catenin stability and WNT signaling and thus promoted memory marker expression in T cells (Gattinoni et al., 2009). The GSK3β effect on differentiation is therefore likely dependent on the context in which GSK3β inhibition occurs. When GSK3β is inhibited early after T cell activation, immunological memory formation is favored, whereas inhibition at a later time point when MVBs are expanded, compromises memory cell generation (Jin et al., 2020). Taken together, lysosomes are involved in regulating T cell size, T cell metabolic status and differentiation by modulating MVB-sequestration and exosomal release of GSK3β.

### Modulation of Late Endosomal Activation of mTORC1

The complex function of mTORC1 in numerous aspects of T cell biology has been the subject of a recent excellent review (Huang et al., 2020). Here, we will focus on the important role of lysosomal and endosomal mTORC1 activity in regulating T cell responses by modulating the balance between anabolic and catabolic metabolism that is essential for effector vs. memory cell fate decision (Araki et al., 2009; Saxton and Sabatini, 2017; Zeng and Chi, 2017; Huang et al., 2020). mTORC1 overactivation by genetic deletion of TSC1 reduced the number of memory precursor CD8^+^ T cells at peak responses after primary infection (Yang et al., 2011; Krishna et al., 2014; Shrestha et al., 2014). In contrast, moderate inhibition of mTORC1 by pharmacological compounds led to increased clonal expansion of antigen-specific T cell memory precursors in primary responses and further enhancement of memory responses after antigen rechallenge in several infection models (Ferrer et al., 2010; Li et al., 2012; Berezhnoy et al., 2014). While lower mTORC1 activation favors TFH and memory development, antigen-primed T cells rapidly induce and maintain a relatively high level of mTORC1 activity before pathogen clearance (Chi, 2012; Ray et al., 2015). This naturally occurring high level of mTORC1 in activated T cells may be important to induce sufficient T effector functions including high proliferation and potent cytokine production.

A recent study proposed a model of how the high level of mTORC1 is maintained in activated T cells in spite of its negative-feedback regulation through downregulating lysosome generation (Jin et al., 2021). In this model, mTORC1 is recruited to late endosomes instead of lysosomes and is activated when lysosomal activity is low (Figure 2). As late endosomes do not have proteolytic activity for protein degradation and amino acid recycling (Piper and Katzmann, 2007), the late endosomal mTORC1 signaling is mediated through the uptake of extracellular free amino acids such as by the leucine transporter SLC7A5, instead of lysosomal recycling (Jin et al., 2021) (Figure 2). Transcription of SLC7A5 is regulated by c-MYC in T cells and therefore upregulated by the reduced lysosomal degradation of c-MYC (Marchingo et al., 2020). Moreover, lysosomal dysfunction expands the maturation of late endosomes, providing a larger platform for mTORC1 activation (Jin et al., 2021). Vam6/Vps39-like protein (VPS39), a member of the HOPS complex, is necessary for late endosome formation and maturation (Rink et al., 2005; Steinauer et al., 2019). VPS39 silencing reduced mTORC1 activity in both human T cells *in vitro* and mouse T cells *in vivo* after antigenic stimulation (Jin et al., 2021). Thus, late endosomal mTORC1 is independent of the negative feedback regulation of lysosomes, and the endosomal pathway may account for the high level of mTORC1 activity in activated T cells in the face of reduced lysosomal activity. Correspondingly, specific inhibition of late endosomal mTORC1 by Vps39 silencing promoted T cell memory formation (Jin et al., 2021). Taken together, reduced lysosomal activity enhances endosomal activation of mTORC1 that contributes to the high mTORC1 activity in primed T cells and thus regulates decisions for T cell fate.

### Mitophagy

As the compartment exerting the final step of degradation, the lysosome is an important regulator of mitochondrial fitness through mitophagy. Mitophagy involves the formation of double-membraned autophagosomes that enclose whole mitochondria or isolated damaged areas, the subsequent fusion with lysosomes, and the ultimate degradation of mitochondria in the autolysosomal lumen (Palikaras et al., 2018; Ma et al., 2020) (Figure 2). Thus, mitophagy prevents intracellular accumulation of dysfunctional mitochondria, promoting mitochondrial fitness. Given the metabolic control of T cell differentiation, mitochondrial fitness is important for the generation of long-lived memory T cells (Gupta et al., 2020; Reina-Campos et al., 2021). In an immunization model, mitophagy promoted memory formation of antigen-specific CD8^+^ T Cells (Gupta et al., 2019). In chronic viral infection models, impaired mitophagy causes T cell dysfunction, at least in part by decreasing mitochondrial biogenesis and inducing robust production of mitochondrial reactive oxygen species (ROS) (Bengsch et al., 2016). In tumor models, accumulation of depolarized mitochondria due to reduced mitophagy induces epigenetic programming of exhaustion (Yu et al., 2020). Mitophagy is also involved in regulating mitochondrial dynamics to sustain mitochondrial fitness in response to metabolic perturbations (Rambold et al., 2011). Mitochondrial architecture remodeling via fusion/fission instructs metabolic adaptations in T cells, determines memory vs. effector cell fate decision, and regulates antitumor immunity (Buck et al., 2016). Taken together, lysosomal dysfunction will influence mitochondrial fitness that is involved in the metabolic regulation of T cell differentiation, in particular in the context of immune aging.

## Lysosomal Dysfunction in T Cell Aging

### Defects in Lysosome Generation After T Cell Activation in Older Adults

The decline of adaptive immunity with age is manifested as the increased susceptibility to newly and previously encountered pathogens and the decreased vaccination efficacy in older individuals (Thompson et al., 2003; Haynes and Swain, 2006; Nikolich-Zugich, 2018). The age-associated defects in T cells arise from imbalanced T cell population homeostasis as well as biased differentiation upon activation (Goronzy and Weyand, 2019), e.g., end-differentiated effector CD45RA^+^ CD28^−^ T cells in older individuals accumulate and exhibit an expression pattern of cytokines and cytotoxic proteins reminiscent of innate immunity and the senescence-associated secretory phenotype (SASP) (Akbar et al., 2016; Callender et al., 2018).

**FIGURE 3:**
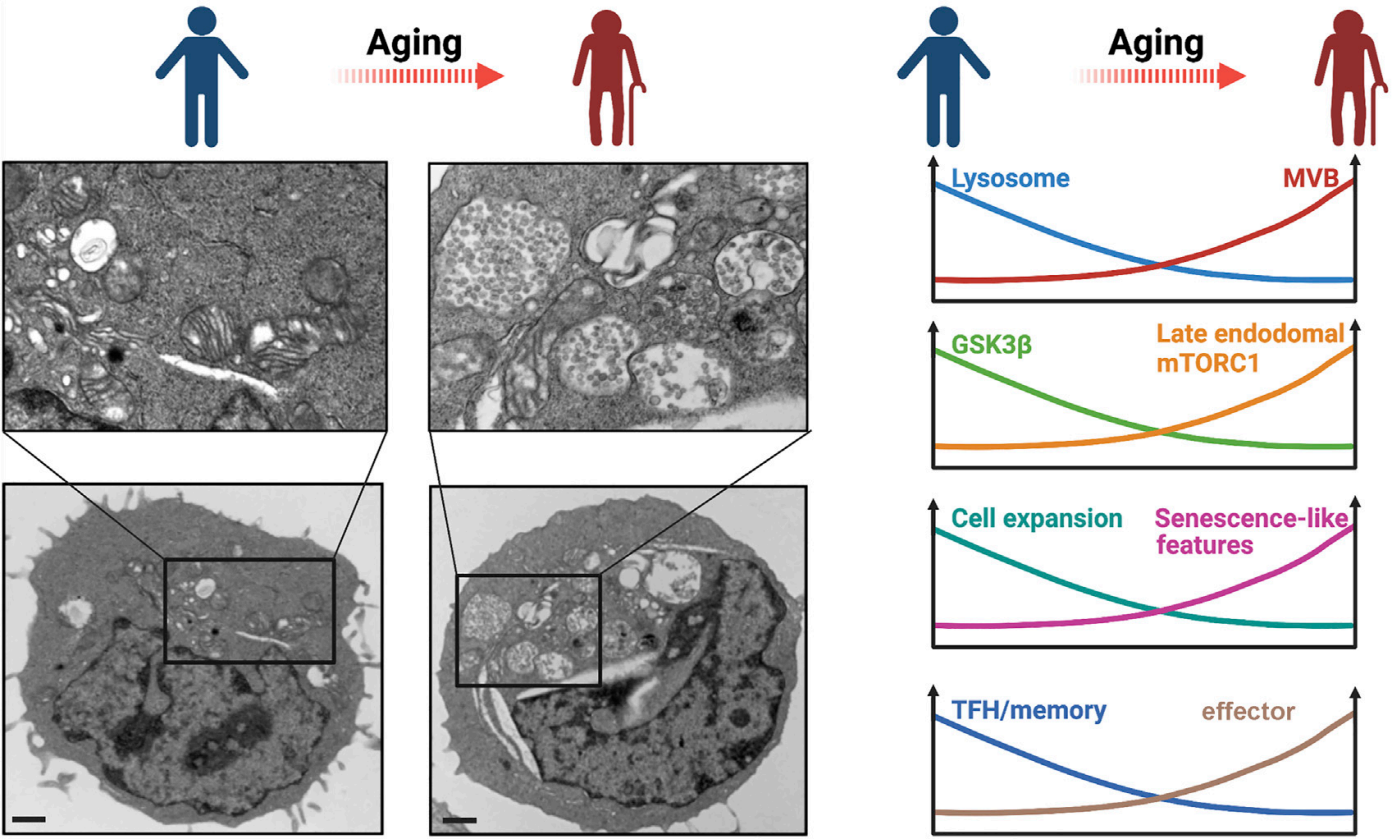
Lysosomal dysfunction in T cell aging. **(Left panel)** Transmission electron microscopy of *in vitro* day 3-stimulated T cells showing MVB enrichment in a T cell from an old adult compared to that from a young adult. Scale bar, 1 μm. Imaged by Jin et al. **(Right panel)** A schematic overview of age-related changes of T cells that are related to reduced lysosomal activity.

Activated T cells from older individuals preferentially differentiate into effector cells with compromised long-term memory cell formation (Kim et al., 2018). To understand the molecular mechanisms underlying the biased differentiation, we systematically compared variables influencing T cell activation in young and older individuals. Naïve T cells from older individuals exhibited elevated AKT activity after activation due to miR-21–mediated reduction of PTEN expression, sustaining effector T cell differentiation (Kim et al., 2018). FOXO1, an AKT-regulated transcription factor important for memory cell generation, is remarkably deficient in activated T cells from older individuals (Jin et al., 2020). FOXO1 is critical in maintaining proteostasis in many cell types by promoting the expression of genes involved in autophagy and the ubiquitin-proteasome system (Webb and Brunet, 2014). In T cells, FOXO1 regulates lysosomal activity through promoting the transcription of TFEB, the master transcription factor for lysosomal genes (Jin et al., 2020). Reduced FOXO1 protein in T cells from older adults causes reduced TFEB and lysosomal gene expression and thus reduced lysosomal proteolytic activities, whereas the formation of MVB, the major source of exosomes, is enhanced (Jin et al., 2020) (Figure 3). MVB expansion in older adults is recapitulated by lysosome inhibition through a pharmacological inhibitor or genetic silencing of TFEB in T cells from young adults (Jin et al., 2020), indicating that reduced lysosomal activity in T cells from older adults accounts for the MVB accumulation.

### Defects in T Cell Proteostasis due to MVB Expansion and GSK3β Sequestration in Older Adults

The consequence of MVB expansion downstream of lysosome-deficiency can be traced with GSK3β. GSK3β is sequestered into MVBs and secreted by exosomes, resulting in reduced intracellular GSK3β activity in activated naïve T cells from older adults (Jin et al., 2020) (Figure 3). Reduced GSK3β leads to increased HK-II translocation to mitochondria and glycolytic flux, driving T cell differentiation into effector cells (Jin et al., 2020) (Figure 3). Impaired lysosomal function promotes the generation of pro-inflammatory T cell subsets and exacerbates *in vivo* inflammatory responses (Baixauli et al., 2015). Reduced GSK3β activity in lysosome-deficient T cells from older adults also promotes intracellular protein stability (Jin et al., 2020), manifested as increased cell mass in T cells with lysosome deficiency induced by *TFEB* silencing (Jin et al., 2020). Loss of proteostasis following autophagy-lysosome inhibition has also been shown to induce increased cell size in other cell types (Miettinen and Bjorklund, 2015). Taken together, loss of cytosolic GSK3β in lysosome-deficient T cells induces an increase in glycolytic capacity, a defect in protein homeostasis accompanied with increased cell size and increased exosome disposal, and a bias of T cell differentiation towards producing pro-inflammatory cytokines and cytotoxic proteins, reminiscent of senescence-associated phenotypes (Jin et al., 2020) (Figure 3). It is likely that the increased cellular protein stability in GSK3β-deficient T cells from older adults not only leads to increased cell size but also to accumulation of damaged proteins that eventually induce cellular senescence. Previous reports have linked GSK3β inhibition to senescence-associated growth suppression (Kim et al., 2013); moreover, GSK3β inhibition–mediated glycogenesis is involved in cell senescence (Seo et al., 2008). Of note, the MVB-dependent release of toxic exosomal granzyme B from T cells in older adults is similar in extent to the lysosome-dependent release from CD8^+^ T cells in young adults and may contribute to inflamm-aging. Taken together, lysosome deficiency after activation is a hallmark of T cell aging that favors differentiation into effector T cells with eventually innate and senescence-like features.

### Defective T Cell Proliferation in Older Adults

Given the role of lysosomes in PD-1 degradation, lysosome-deficient T cells from older adults may have impaired proliferation upon antigen stimulation. Indeed, naïve T cells from older adults showed reduced proliferation and expansion after anti-CD3/anti-CD28 stimulation in the presence of PD-L1 (programmed death-ligand 1)–Fc cross-linked with anti-human immunoglobulin G (Jin et al., 2021) (Figure 3). In contrast, in the absence of exogenous PD-L1-Fc, age-related proliferation differences were not observed, supporting the notion that T cells from older adults only exhibit proliferative defects upon receiving inhibitory signals from PD-1. PD-1 blockade also significantly improved T cell proliferation from older adults in response to pathogen-derived peptides presented by antigen-presenting cells (Jin et al., 2021). Enhanced proliferation was recapitulated when lysosomal function was improved through silencing of VPS39 (Jin et al., 2021). Similarly, Foxo1-deficient mouse T cells that mimic TFEB- and lysosome-deficient T cells, showed up to a fourfold increase of cell surface PD-1 protein levels and a dramatic decline of antigen-specific cell proliferation after antigen priming *in vivo* (Stone et al., 2015), consistent with our *in vitro* human cell data. These data support the notion that inhibitory signals from increased expression of PD-1 in older adults curtails the acute response of T cells that are otherwise functional and not exhausted.

In addition to PD-1, lysosomes are involved in the CMA-mediated degradation of E3 ubiquitin-protein ligase Itchy homolog (Itch) and the calcineurin inhibitor Rcan-1, two negative regulators of TCR signaling (Valdor et al., 2014). CMA is impaired in T cells of both older humans and mice resulting in the accumulation of these inhibitors and impaired T cell activation and proliferation (Valdor et al., 2014).

### Impaired TFH and Memory Responses in Older Adults

Generation of T follicular helper (TFH) and T memory cells is at the core of successful vaccination. mTORC1 activation is one of the major determinants in T cell fate decision, favoring effector over memory or TFH cells (Araki et al., 2009; Ray et al., 2015). mTORC1 activation was shown to be more sustained in the T cell response of older individuals (Kim et al., 2018) (Figure 3). Surprisingly, mTORC1 signaling in activated T cells from older adults occurs preferentially at the late endosome rather than at the lysosome (Jin et al., 2021). This has important consequences in the regulation of the mTORC1 activity as it switches a negative regulatory feedback to a feedforward loop that propagates mTORC1 activation. In the negative feedback loop, mTORC1 phosphorylates TFEB, thereby inhibiting the generation of lysosomes where mTORC1 activation normally occurs (Roczniak-Ferguson et al., 2012). However, reduced lysosomal activity in T cells from older adults induces the expansion of late endosomes and the increased expression of the plasma membrane leucine transporter SLC7A5 that promotes extracellular leucine uptake (Jin et al., 2021). Increased late endosomal mass and increased SLC7A5 activity support further mTORC1 activation, leading to high and sustained mTORC1 activity and further inhibition of TFEB in T cells (Jin et al., 2021). Overactivation of late endosomal mTORC1 impairs memory T cell formation leading to both reduced number and quality of memory T cells (Jin et al., 2021) (Figure 3). Inhibition of late endosomal mTORC1 by silencing VPS39 restores lysosomal activity and TFH and memory generation in T cells from older humans and augments the generation of germinal centers and CD8^+^ memory responses to LCMV infection in mice (Jin et al., 2021). Moreover, as discussed above, loss of GSK3β during late activation also inhibits memory T cell responses through promoting HK-II mediated upregulation of glycolytic flux (Jin et al., 2020). In addition, persistence of dysfunctional mitochondria in T cells due to defective mitochondrial turnover by the autophagy/lysosome pathway may contribute to the impaired memory T cell generation in older individuals (Bektas et al., 2019). Overall, reduced lysosomal activity in T cells from older individuals impairs TFH and memory T cell responses by reversing the negative feedback loop that controls mTORC1 activity, by reducing cytosolic GSK3β due to enhanced sequestration into MVBs and by promoting intracellular accumulation of dysfunctional mitochondria.

Impaired TFH and T memory generation does not mean improved effector T cell responses in older individuals. While T cells from older adults favor to differentiate into effector cells that are functional in terms of cytokine production *in vitro*, the T cell effector arm is compromised in older individuals by independent mechanisms. For example and as discussed above, PD-1 overexpression due to lysosomal dysfunction impairs T cell proliferation. Independent of lysosomal defects, proliferating T cells from older adults exhibit replication stress and increased DNA damage responses, in part due to reduced upregulation of histone transcription (Jin et al., 2021; Kim et al., 2021). Finally, increased expression of BLIMP1 in T cell responses of older adults induces CD39 expression by antagonizing BCL6 (Cao et al., 2020; Zhang et al., 2021). The associated increased adenosine production is immunosuppressive and pro-apoptotic (Fang et al., 2016).

## Concluding Remarks

Most past studies investigated the role of lysosomes in lysosomal degradation-specialized cells such as macrophages and dendritic cells (Jakos et al., 2020). Studies exploring the function of lysosomes in the degradation-nonspecialized cells such as those mounting an adaptive immune response are few but document that the lysosome is emerging as an important player in regulating T cell immunity in several dimensions: 1) conferring cytotoxic activity to CD8^+^ T cells: 2) improving antigen-specific T cell expansion by degrading PD-1: 3) modulating MVB mass and secretion of exosomes; 4) reducing cell size and effector cell generation by inhibiting the MVB-mediated flux of GSK3β; 5) suppressing late endosomal mTORC1 activation and thus promoting memory generation; 6) improving mitochondrial fitness and T cell function by supporting mitophagy. Impaired lysosomal function in T cells from older adults affects most of these dimensions, and particularly so through the expansion of the late endosomal compartment. Several of the age-related T cell defects such as their inflammatory and senescence-like features with reduced proliferative potential and impaired TFH and memory development can at least in part be attributed to relative lysosomal deficiency. Our current knowledge on lysosomes in T cells is still limited, leaving many questions to be answered. In particular, how can we boost lysosomal activity to enhance T cell function? Most currently known lysosome modulators are pharmacological inhibitors and not activators. How can we interfere with the dysregulated signaling network in older T cells that leads to reduced lysosomal biogenesis and expansion of the MVB compartment, with far-reaching consequences for cellular proteostasis and metabolic regulation? A better understanding of how lysosomes regulate T cell immunity is needed to enable the design of interventions that could improve T cell immunity in older age.
